# Selection and Validation of Reference Genes for Quantitative Real-Time PCR in Buckwheat (*Fagopyrum esculentum*) Based on Transcriptome Sequence Data

**DOI:** 10.1371/journal.pone.0019434

**Published:** 2011-05-12

**Authors:** Natalia V. Demidenko, Maria D. Logacheva, Aleksey A. Penin

**Affiliations:** 1 Department of Genetics, Biological Faculty, M.V. Lomonosov Moscow State University, Moscow, Russia; 2 Department of Evolutionary Biochemistry, A.N. Belozersky Institute of Physico-Chemical Biology, M.V. Lomonosov Moscow State University, Moscow, Russia; 3 Evolutionary Genomics Laboratory, Faculty of Bioengineering and Bioinformatics, M.V. Lomonosov Moscow State University, Moscow, Russia; 4 A.A. Kharkevich Institute for Information Transmission Problems, Russian Academy of Science, Moscow, Russia; National Institutes of Health, United States of America

## Abstract

Quantitative reverse transcription PCR (qRT-PCR) is one of the most precise and widely used methods of gene expression analysis. A necessary prerequisite of exact and reliable data is the accurate choice of reference genes. We studied the expression stability of potential reference genes in common buckwheat (*Fagopyrum esculentum*) in order to find the optimal reference for gene expression analysis in this economically important crop. Recently sequenced buckwheat floral transcriptome was used as source of sequence information. Expression stability of eight candidate reference genes was assessed in different plant structures (leaves and inflorescences at two stages of development and fruits). These genes are the orthologs of *Arabidopsis* genes identified as stable in a genome-wide survey gene of expression stability and a traditionally used housekeeping gene *GAPDH*. Three software applications – geNorm, NormFinder and BestKeeper - were used to estimate expression stability and provided congruent results. The orthologs of *AT4G33380* (expressed protein of unknown function, *Expressed1*), *AT2G28390* (SAND family protein, *SAND*) and *AT5G46630* (clathrin adapter complex subunit family protein, *CACS*) are revealed as the most stable. We recommend using the combination of *Expressed1*, *SAND* and *CACS* for the normalization of gene expression data in studies on buckwheat using qRT-PCR. These genes are listed among five the most stably expressed in *Arabidopsi*s that emphasizes utility of the studies on model plants as a framework for other species.

## Introduction

Analysis of gene expression using qRT-PCR is widely used in many fields of plant research including development [1], [2], response to abiotic stress [3], [4], and to pathogen infection [5], [6]. It remains the most sensitive and most accessible for wide range of researchers compared with other methods of gene expression analysis (microarray, Northern blot, whole transcriptome shotgun sequencing). Recently a set of guidelines aimed at the improvement of the reliability and reproducibility of the studies using qRT-PCR was published [7]. Despite its numerous advantages, this method has however several issues, with one of the most important being the normalization. There are several strategies of normalization [8] but the most popular is the use of reference gene. This is a gene whose expression is presumably stable in control and experimental conditions. Though there are several genes traditionally used for this purpose (*18S rRNA*, *actin*, *tubulin*), many studies demonstrate that the expression of these genes varies greatly and the selection of alternative genes is favourable for accurate normalization [9], [10].

In this article we report the search and validation of reference genes for quantitative real-time PCR in buckwheat (*Fagopyrum esculentum*). Buckwheat is an important grain and honey crop widely cultivated in several countries (Canada, China, Japan, Russia and Ukraine). Its seeds are used as whole grain, groats and flour. Because of similar usage, buckwheat is usually classified as cereal. However, phylogenetically it is far from true cereals - members of the monocot family Poaceae. Buckwheat belongs to the family Polygonaceae from the order Caryophyllales - the group of flowering plants which is distant from any model plant species. By now, only few studies investigating buckwheat gene expression by real-time PCR are reported [e.g.11], [ 12].

We have evaluated the expression stability of eight candidate reference genes in five developmental stages in *Fagopyrum esculentum*. These genes are putative orthologs of the *Arabidopsis* genes identified as the most stable in [9]; seven of them are not widely used as reference and one is a traditionally used housekeeping gene, that of *glyceraldehyde 3-phosphate dehydrogenase*. To obtain the sequences of these genes we used the assembly of buckwheat floral transcriptome sequenced by 454 technology [13].

Large-scale sequencing of buckwheat genes reported in [13] allows efficient search of candidate genes for agriculturally important traits. Validation of reference genes reported in this study will enable the analysis of their expression in a variety of developmental and environmental conditions.

## Results

### Selection of candidate reference genes and amplification specificity and efficiency

**Figure 1:**
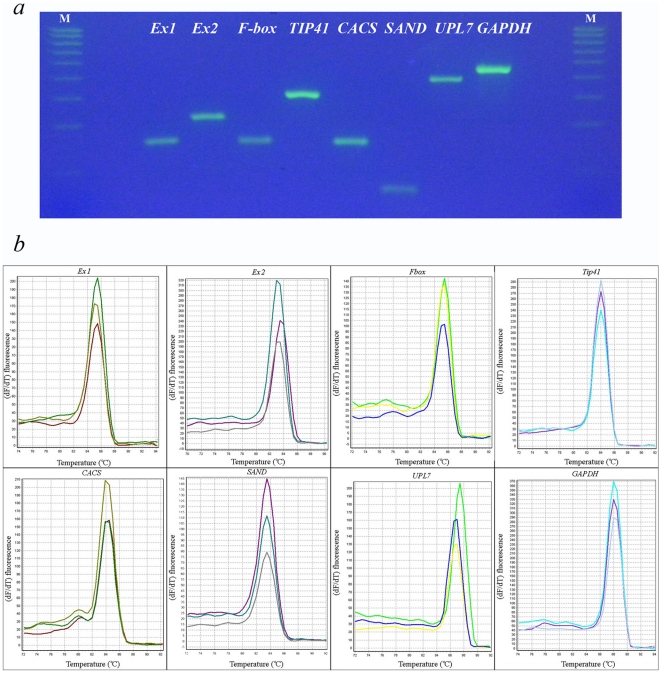
Specificity of RT-qPCR and amplicon length. (a) amplified fragments were separated by 3% SYBR Green staining agarose gel. Highest marker band corresponds to 500 bp; each following band is 50 bp less than preceding (b) melting curves generated for all amplicons.

First, we performed a search for the orthologs of the genes listed as stably expressed in the genome-wide investigation of *Arabidopsis thaliana* [9] in buckwheat transcriptome data. This search revealed eight genes for which only one sequence with high similarity to a certain gene was found. These genes are the orthologs of *AT4G33380* (unknown function, *Expressed1*), *AT4G26410* (unknown function, *Expressed2*), *AT5G15710* (F-box family, *F-box*), *AT4G34270* (Tip41-like, *Tip41*), *AT5G46630* (clathrin adaptor complex subunit, *CACS*), *AT2G28390* (SAND family, *SAND*), *AT3G53090* (ubiquitin protein ligase 7, *UPL7*) and *AT1G13440* (glyceraldehyde 3-phosphate dehydrogenase, *GAPDH*). For other genes, either no hits or multiple sequences that have high similarity to the *Arabidopsis* gene but differ significantly one from another were found. Thus these eight genes were chosen as candidate reference genes and used for primer design. Agarose gel electrophoresis with SYBR Green staining and melting curve analysis revealed single products of expected length (Fig. 1a, b). The sequencing of amplicons confirmed that single products corresponding to the contigs that were used for primer design are generated (GenBank accession numbers: JF343809 - *Expressed1*, JF343807 - *Expressed2*, JF343804 - *F-box*, JF343806 - *Tip41*, JF343811 - *CACS*, JF343810 - *SAND*, JF343805 - *UPL7* and JF343808 - *GAPDH*). No primer dimers or non-specific amplicons were revealed with no template amplification. No signals were also detected from no RT-controls, what reveals no genomic DNA contamination. Average amplification efficiency varied from 89,1% and 91,3% (Table 1).

**Table 1:** Selected candidate reference genes, primers and different parameters derived from qRT-PCR analysis.

Name	Primer sequences (forward/reverse)	Amplicon length (bp)	Presence of PCR product with genomic DNA used as template	Amplification efficiency
Expressed1	AGGCCAGTTCCTGCTGAATGTAATGC	127	no	1,891
	TAGCCTGATCCAAACAAGCCTGGCAA			
Expressed2	ACTCTGGGAAGATTTCAAAGTGAGGAGACA	159	yes	1,898
	CTCAGTGCGACCCTGTTGCATTTCTT			
Fbox	TCGAGGTTGTATCTGGAAACTTTGTCGC	125	no	1,906
	CCGGCAACTAAATACCCATCCAAGAGAG			
Tip41	CGATACTGGCTTAGAGTTGATGGTGTGC	200	no*	1,892
	GGAAGCCTCTGATTGATGATGCTTGGA			
CACS	AAGACAGTCAGTTTCGTGCCACCTGA	125	yes	1,891
	TCCATGCGTGTTCTACCCAACTCCTT			
SAND	GACCCCCTTGCAGACAAAGCATTGGCA	79	yes	1,830
	TCTCGTTCTCAACGTCTTTTACCCACTGG			
UPL7	TGGTTACACTGAGGGAAGTCGCACTGTT	241	yes	1,899
	ATTGTAGCAGGTGGAAGCTGAAGGAAGC			
GAPDH	AGTTGCACTACCAACTGCCTTGCT	261	no	1,913
	AGGTCAACCACGGACACATCAACA			

*two products can be obtained if genomic DNA is used as template.

### Expression stability of candidate reference genes

**Figure 2:**
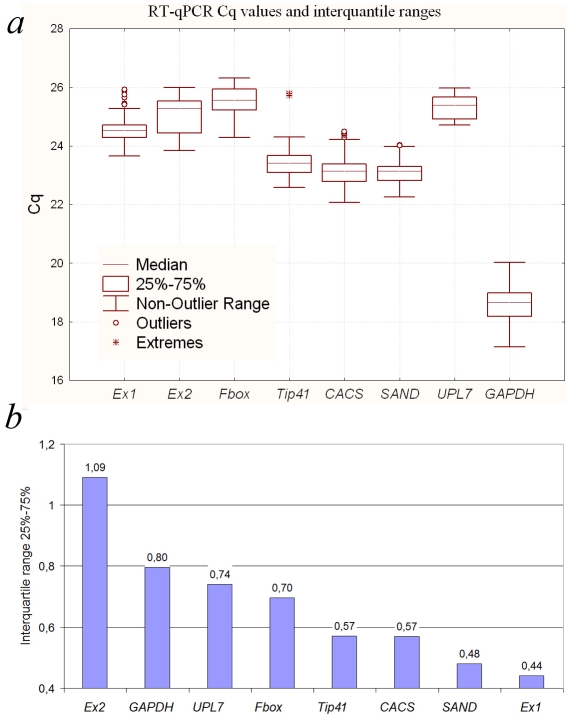
RT-qPCR Cq values and interquantile ranges. (A) Cq values for each reference gene in all buckwheat samples. A line across the box depicts the median. The box indicates the 25% and 75% percentiles. Whiskers represent the maximum and minimum values, circles represent outliers and asterisks indicate extremes. (B) interquantile ranges indicates variability of Cq values among 25% and 75%.

Quantification cycle (Cq) data were obtained from each reaction with eight primer pairs. To reveal differences in transcript levels between various candidate genes, the average Cq was calculated across all samples (Fig. 2a). For seven genes Cq values ranged between 23 and 25 cycles, and only one gene – *GAPDH* – have the most abundant transcript level (mean Cq = 18,6). To determine genes with the least Cq values dispersal interquantile ranges were calculated. There are four genes with relatively low Cq dispersal: *Expressed 1*, *SAND*, *CACS* and *Tip41* (Fig. 2b).

Three programs were applied to calculate the expression stability of candidate reference genes: geNorm [14], NormFinder [15] and BestKeeper [16]. Expression stability values were determined across all samples. Cq values were used directly for stability calculations for BestKeeper or were transformed to relative quantities using delta-Cq method (geNorm, NormFinder).

#### a) GeNorm analysis

**Figure 3:**
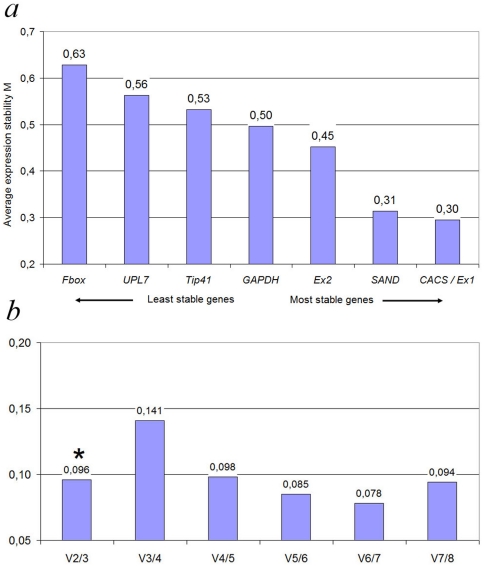
geNorm ranking of candidate reference genes and pairwise variation (V) to determine optimal number of reference genes. (A) expression stability of all 8 candidate reference genes from all samples. A lower average expression stability M value indicates more stable expression. (B) pairwise variantion calculated by geNorm to determine the minimum number of reference genes for accurate normalisation. Asterisk indicates the optimal number of reference genes.

GeNorm is a Visual Basic application tool for Microsoft Excel that operates on the assumption that expression ratio of two ideal reference genes is constant throughout the different groups of templates. Gene expression stability value (*M*) was calculated for all genes involved into investigation. Vandesompele *et al.* [14] recommends using *M* value below the threshold of 1.5. In our analysis all genes had *M* less than 1,5 that allows to consider genes as rather stable. Three genes: *SAND*, *Expressed1* and *CACS* had the highest expression stability values (the lowest *M* value), *F-box* revealed least stability value and other four genes occupy the intermediate positions between these both groups (Fig. 3a). To determine optimal number of reference genes geNorm calculates the pairwise variation V*_n_*/V*_n_*_+1_ between two sequential normalization factors NF*_n_* and NF*_n_*_+1_ that contain an increasing number of reference genes. The large value of variation means that adding another gene is necessary for calculation of a reliable normalization factor. The most accurate way is to include the next gene until the variation V*_n_*/V*_n_*_+1_ drops below recommended threshold of 0.15 and current investigation revealed that pairwise variation V_2/3_ was 0.096, that indicates, that addition of the second gene is necessary for accurate analysis and adding the third gene is optional (Fig. 3b).

#### b) NormFinder analysis

**Figure 4:**
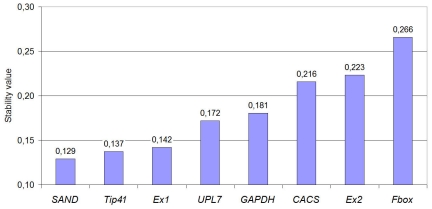
NormFinder ranking of reference genes. Ranking of reference genes based on their stability value, calculated for all samples divided into five groups (fruits, leaves and inflorescences on various developmental stages).

NormFinder is another Excel application, which uses a model-based approach for identifying the most stable reference genes, based on combining samples into groups. In current study these five groups correspond to different types of samples (fruits, leaves and inflorescences on various developmental stages). NormFinder calculates intra- and inter-group variations and genes with the least ones are considered to be stable. As well as geNorm, NormFinder revealed *SAND* and *Expressed1* as stable, but third gene was *Tip41*, while geNorm ranks it only on the sixth place (Fig. 4). However NormFinder results suggest using as references the same genes as geNorm results. Similar to geNorm, NormFinder revealed that the least stable gene is *F-box*.

#### c) BestKeeper analysis

**Figure 5:**
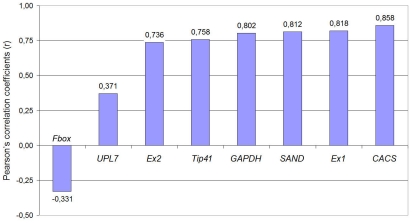
BestKeeper ranking of reference genes. Statistic calculations of genes stability based on correlations between reference genes and BestKeeper Index. Values of Pearson's correlation coefficients (r) showed in the figure.

Third applet uses raw Cq data and determines the most stably expressed genes based on coefficient of correlation (r) to the BestKeeper Index (BI). BI is the geometric mean of Cq values of best reference genes. The BestKeeper revealed, that the best correlations were obtained for *CACS* (r = 0.858), *Expressed1* (r = 0.818) and *SAND* (r = 0.812) with p value of 0.001. *F-box* ranked as the least stable gene with r = −0.331 (Fig. 5).

### The effects of choice of reference gene

**Figure 6:**
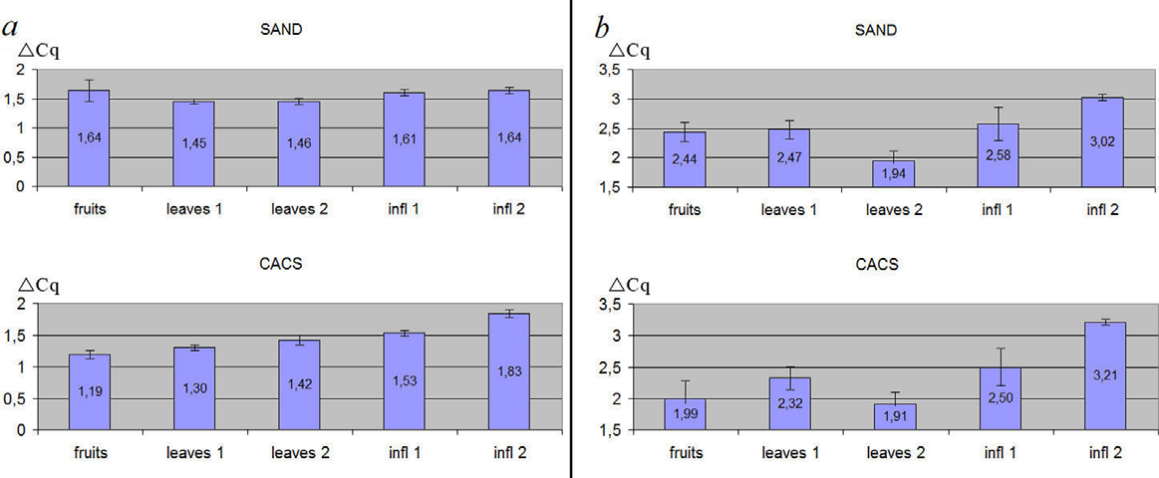
The relative expression level of reference genes in buckwheat. (a) two most stable genes – *SAND* and *CACS* normalised by *Expressed1*, (b) *SAND* and *CACS* normalised by *F-box*. Data was obtained from all sample groups (fruits, leaves and inflorescences on various developmental stages). Error bars represent standard error of the mean.

To illustrate effects of the choice of non-optimal reference gene we modeled a situation of gene expression analysis, taking as reference in the first case the gene identified as the most stable (*Expressed1*) and then the least stable (*F-box*). Two other genes, identified as stable - *SAND* and *CACS* - were considered as target genes. When the expression level of *SAND* and *CACS* was calculated relative to *Expressed1* it was found to be stable (1.4–1.6 for *SAND* and 1.2–1.8 for *CACS*) in all plant structures (Fig. 6a). In contrast, when *F-box* was used as reference, the relative expression of these genes varied greatly: 1.9–3 for *SAND* and 1.9–3.2 for *CACS* (Fig. 6b). This contradicts to the results of evaluation of gene expression stability (see above) that demonstrate the stability of expression of *SAND* and *CACS* in different buckwheat tissues. Thus great difference between gene expression levels of *SAND* and *CACS* is an artifact caused by the wrong choice of reference gene.

## Discussion

### Transcriptome sequence data as a source for robust normalization genes

Normalization is one of the key factors affecting the accuracy and reliability of the quantitative gene expression analysis. In view of this, the systematic validation of reference genes for new experimental systems was advocated [17] including in plant science [10], and several algorithms for the selection of the most stably expressed genes were developed [14]–[16]. These algorithms became now very widely used - for example, geNorm – the Excel plug-in, implementing one of them was downloaded about 15 thousand times [18]. The number of articles reporting the validation of reference genes in plants also increased in past few years [19]–[21]. Most of them however explore the stability of traditionally used housekeeping genes such as those encoding 18S ribosomal RNA, ribosomal proteins, actins, tubulins, elongation factor 1alpha, GAPDH and so on (Table S1). In a genome-wide survey of gene expression stability in *Arabidopsis thaliana* it was shown that many genes are more stably expressed than these reference genes [9]. These genes are now widely adopted for normalization in the studies on *Arabidopsis* [22], [23]. The orthologs of these genes are obvious candidates for reference genes in other species; they were, however, tested only in few species [24], [25] and in all these species they were found to be among the most stably expressed (Table S1).

One of the main reasons of the limited use of these genes for normalization in other species is the limited availability of their sequences. While highly conserved genes such as *actin*, *ef1-alpha*, *18S rRNA* can be easily sequenced using consensus primers (or even consensus primers can be used for qPCR [26]) the sequences of these novel reference genes are variable between species that complicates the design of primers for sequencing or qPCR. Also most of them are expressed at much lower levels than traditional reference genes thus they are less likely to be represented in EST sequencing data (especially low-coverage). With the advent of next-generation sequencing, large-scale transcriptomic data became available for many plant species [27]–[29] and these data presumably have a great potential as a source of candidate reference genes. However to our knowledge they have never been used for this purpose. Our results demonstrate that 454 transcriptome sequencing data are indeed a useful source of potential reference genes.

### The choice of reference gene for real-time PCR data normalization in buckwheat

Our evaluation of the stability of candidate reference genes using three different applications yielded similar results. In particular, *Expressed1* and *SAND* were listed among as best reference genes. Since these applications use principally different algorithms for their calculations it is common to get different results [30], [26]. The few discrepancies that are however observed is that *CACS* was considered as stable by only two programs and *TIP41* is highly ranked by NormFinder, in contrast to two other programs. *F-box* was revealed as the worse gene by all three approaches (Table 2). This is consistent with the genome-wide investigation [9] where these genes are among five the most stable and supports the suggestion that the orthologs of the novel reference genes that they have identified could serve the same purposes in other species. Similar results were obtained in the study of gene expression stability in tomato [24] – one of the few studies adopting new reference genes proposed in [9]. Optimal number of reference genes calculated by geNorm is two – *Expressed1* and *CACS*. However taking into account that NormFinder does not support high stability of *CACS* but lists *SAND* as the most stable (according to two other applications *SAND* is on the third place) and that *CACS* expression is known to be affected by several treatments [31] we suggest using three reference genes - *Expressed1*, *CACS* and *SAND*. We expect that use of these genes for normalization in the future studies evaluating gene expression in buckwheat using qRT-PCR will improve the sensitivity and reproducibility of the results. In contrast, the choice of non-optimal reference gene can lead to inaccurate results.

**Table 2:** General range of reference genes, based on interquantile ranges, geNorm, NormFinder and BestKeeper analysis.

	←least stable				most stable→			
Interquantile ranges	*Expressed2*	*GAPDH*	*UPL7*	*Fbox*	*Tip4*	*CACS*	*SAND*	*Expressed1*
GeNorm	*Fbox*	*UPL7*	*Tip4*	*GAPDH*	*Expressed2*	***SAND***	***Expressed1/CACS***	
NormFinder	*Fbox*	*Expressed2*	***CACS***	*GAPDH*	*UPL7*	***Expressed1***	*Tip4*	***SAND***
BestKeeper	*Fbox*	*UPL7*	*Expressed2*	*Tip4*	*GAPDH*	***SAND***	***Expressed1***	***CACS***

**Less stable genes are marked in** ***italic*** **, those that are ranked among three the most stable genes in all four approaches are given in** *underlined bold italic*, **genes ranked as stable by three of four approaches are marked in** *bold italic*.

### Conclusions

First, this study demonstrates that the data derived from 454 sequencing of normalized cDNA libraries are useful as a source of candidate reference genes. We have evaluated the expression stability of 8 candidate reference genes in different organs of common buckwheat using three software applications – geNorm, NormFinder and BestKeeper. Despite small incongruences between different algorithms they all indicate on the stability of three buckwheat genes - the orthologs of *AT4G33380*, *SAND* and *CACS*. According to geNorm, the combination of *Expressed1* and *CACS* is sufficient to provide accurate normalization however we suggest include also *SAND* in the set of reference genes for buckwheat. These results can be useful for the studies on gene expression in buckwheat using qRT-PCR. Interestingly, that these genes are listed among five the most stably expressed in *Arabidopsis*. This supports the applicability of the data inferred from the study of model plants to the non-models ones and encourages their use as a framework when performing the reference gene normalization experiments in other species.

## Materials and Methods

### Plant material and biological samples

Samples were collected from the buckwheat plants grown in a greenhouse at 20–25°C and relative humidity 80% under long day (16 hours light/8 hours dark) conditions. Five samples were taken: 1) fruits; 2) leaves with the developing leaf blade; 3) mature leaves; 4) developing inflorescences (5–8 mm in length) 5) inflorescences on the stage of the anthesis of first flower. All samples were collected in two replicates.

### RNA extraction and cDNA synthesis

Total RNA was isolated from 80
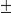
5 mg of plant material using RNeasy Plant Kit (Qiagen, USA) according to the modified protocol [32], based on CTAB DNA extraction protocol [33] with further use of the RNeasy Plant Mini Kit (Qiagen, Stanford, California, USA). To eliminate genomic DNA from RNA samples they were treated twice with RNase-Free DNase (Qiagen, USA). First digestion was performed according manufacturer's instructions then columns were washed with 350 µl of RW1 and digestion was repeated. To evaluate RNA integrity RNA was visualized on 1% SYBR Green stained agarose gel. Clear bands corresponding to 18S and 28S rRNA and absence of smear were observed indicating minimal degradation of RNA. Concentration and purity of total RNA were determined with NanoDrop1000 spectrophotometer (Thermo Scientific, USA). Samples with concentration more than 100 ng/µl and optical density absorption ratio A260/A280 more than 1.8 were taken for cDNA synthesis. Individual RNA samples were stored at −70°C with the addition of RNAse inhibitor RNasin (Sileks, Russia) and then were adjusted to the concentration of 100
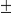
5 ng/µl for reverse transcription. First strand cDNA was performed using “First strand cDNA synthesis kit” (Sileks, Russia) with 24T primer (0,4 nmol per reaction) in 25 µl reaction mix according manufacturer's protocol. Before each qPCR stage cDNA samples were heated (65°C–90 seconds 40°C–30 seconds) and then cDNA products were diluted 10-fold prior to use in real-time PCR.

### Primer design and PCR conditions

Based on the transcriptome data sequences of eight potential reference genes were obtained. Buckwheat transcriptome database was searched with TBLASTX (e-value 10^−6^) using the sequences of *Arabidopsis* genes from genome-wide investigation of *A.thaliana* [9] as query. For all genes primer pairs were designed using PrimerQuest [34] with the following parameters: optimal length 25 nucleotides, melting temperature 60–65°C, product size range 79–261 base pairs, maximum self complementarity at 3′end – 3 nucleotides and then checked for the absence of stable hairpins and dimers using OligoAnalyser [35]. To check primer specificity real-time PCR was performed on cDNA and products were analyzed on 3% SYBR Green staining agarose gel and by melting curves. Amplification efficiency was accessed using Miner 2.2 software [36], [37].

Real-time PCR reactions were run on ANK-32 thermocycler (Syntol, Russia) using 2.5× RT-PCR reaction mix (Syntol, Russia). To detect dsDNA synthesis EvaGreen dye was used since it is known to have lower inhibitory effect on PCR than commonly used SYBR Green [38]. Each reaction was performed in 25 µl mix containing 400 nmol of each primer and 1 µl of 1∶10 diluted cDNA. The following amplification program was used: initial denaturation 95°C for 5 minutes, then 35 cycles of 95°C at 15 seconds and 62°C at 60 seconds. Each sample was analyzed in three technical replicates; mean Ct values were calculated. Mean Ct dispersal for technical replicates did not exceed 0,3 cycle. To ensure the absence of contamination or primer dimer formation for each primer pair non-template control reaction was run. Reverse transcription negative control was also included to ensure absence of genomic DNA in the template. These no-RT control reactions were run with primers to *AT4G33380* ortholog as these primers anneal within one exon. To confirm the specificity of primer annealing the amplicons corresponding to each primer pair were sequenced. DNA sequencing was performed in the interinstitutional sequencing center at Engelhardt Institute of Molecular Biology (Moscow, Russia) using ABI PRISM BigDye Terminator kit v. 3.1 with following analysis on ABI PRISM 3730 genetic analyzer (Applied Biosystems, USA). Sequences of the amplicons were deposited in the GenBank (accession numbers are listed in the Results section).

### Analysis of gene stability

Gene expression stability was estimated with three various approaches: geNorm (ver. 3.5) [14], NormFonder [15] and BestKeeper [16]. Real-time PCR Ct values were transformed into Cq by standard formula: 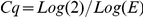, where E is the efficiency of the amplification of each primer pair. Then for both programs Cq values were transformed to relative quantities using the delta-Cq formula 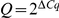, where ΔCq is the difference between the sample with lowest Cq and the Cq value of the sample of question.

The geNorm statistical algorithm is based on pairwise variation of a single reference candidate gene relative to all other genes were taken into investigation. The main assumption of this approach is that the expression ratio of two ideal reference genes is identical in all samples regardless of tissue type. In this case variation of the ratios of two candidate reference genes reflects instability in expression levels of one gene. Then reference expression stability measure (*M* value) is calculated with the average of pairwise variations. Vandesompele *et al.* recommend use only those genes, which *M* value does not exceed 1,5. Genes with lowest *M* value are the most stable and geNorm estimates the normalization factor (NF*_n_*) using geometric mean of expression levels of *n* best reference genes. Finally geNorm defines an optimal number of reference genes, because there are no reasons to use whole genes with low variability if NF doesn't significally change.

Another model-based approach – NormFinder – determines expression stability of candidate references according to their group origin (tissue, organ etc.) and compares expression variation between groups. With help of given group identifier the program divide data to panels, which may be differ due to experimental conditions: inflorescence, leaf, root etc. The main goal of the approach is to determine inter- and intra-group variation and combine both results in a stability value for each investigated gene. According to this algorithm genes with lowest stability will be top ranked.

Third program – BestKeeper – calculates standard deviation (SD) and the coefficient of variation (CV) based of Cq values of all reference candidate genes. Genes with SD less than 1 are considered to be stable. Then program calculates a pairwise correlation coefficient between each gene and the BestKeeper index – geometric mean between Cq values of stable genes grouped together. Genes with the highest coefficient of correlation with the BestKeeper Index indicates the highest stability.

## Supporting Information

Table S1 **Summary of the results of the studies reporting reference gene validation in plants.** This table lists the species for which reference gene validation was reported, the candidate reference genes, the programs used, the gene(s) inferred as the most stable and the corresponding citation. The studies reporting validation of reference gene in some particular conditions (for example, under pathogen infection) are not included.
(DOC)
