# Supplementary material for: Selection and Validation of Reference Genes for Quantitative Real-Time PCR in Buckwheat (Fagopyrum esculentum) Based on Transcriptome Sequence Data
Source: PLoS One. 2011 May 12;6(5):e19434. doi: 10.1371/journal.pone.0019434 (PMC3093374; doi:10.1371/journal.pone.0019434)
Supplement: Table S1 — Summary of the results of the studies reporting reference gene validation in plants. This table lists the species for which reference gene validation was reported, the candidate reference genes, the programs used, the gene(s) inferred as the most stable and the corresponding citation. The studies reporting validation of reference gene in some particular conditions (for example, under pathogen infection) are not included. (DOC) [file pone.0019434.s001.doc]

### Table S1. Summary of the results of the studies reporting reference gene validation in plants.

This table lists the species for which reference gene validation was reported, the candidate reference genes, the programs used, the gene(s) inferred as the most stable and the corresponding citation. The studies reporting validation of reference gene in some particular conditions (for example, under pathogen infection) are not included.

| Object | Candidate reference genes | Ranking order | | | Best genes | Reference |
| --- | --- | --- | --- | --- | --- | --- |
|  |  | geNorm | NormFinder | BestKeeper |  |  |
| *Cicer*  *arietinum* | *ACT1*  *EF1α*  ***GAPDH***  *IF4α*  *TUB6*  *UBC*  *UBQ5*  *UBQ10*  *18sRNA*  *25sRNA*  *HSP90*  *GRX* | *EF1α/ HSP90*  *IF4α*  *GAPDH*  *UBC*  *25sRNA*  *18sRNA*  *UBQ10*  *UBQ5*  *TUB6*  *GRX*  *ACT1* |  |  | EF1α  HSP90 | 1 |
| *Nicotiana*  *tabacum* | *18SrRNA*  Actin*(Tac9)*  *EF1α*  ribosomal protein L25  *α-*Tubulin *(tubA1)*  *β-*Tubulin  *Ntubc-2*  2A (PP2A) | *EF1α/L25*  *Ntubc-2*  PP2A  *18SrRNA*  Actin  *β-*Tubulin  *α-*Tubulin | PP2A  *18SrRNA*  *Ntubc-2*  *L25*  Actin  *β-*Tubulin  *α-*Tubulin |  | *L25*  *EF1α*  *Ntubc-2* | 2 |
| *Gossipium*  *hirsutum* | GhACT4  GhEF1α 5  GhPP2A1  GhMZA  GhPTB  GhGAPC2  GhβTUB3  GhUBQ14  GhFBX6 | GhPP2A1/  GhPTB  GhMZA  GhUBQ14  GhACT4  GhEF1α 5  GhFBX6  GhβTUB3  GhGAPC2 | GhPP2A1  GhUBQ14  GhACT4  GhMZA  GhPTB  GhEF1α 5  GhFBX6  GhβTUB3  GhGAPC2 |  | GhPP2A1  GhUBQ14 | 3 |
| Soybean 1 | ACT11  ACT2/7  CYP  EF1β  TUA5  TUB4  UBQ10  HDC  SKIP16  MTP  PEPKR1  **TIP41**  UKN1  UKN2 | SKIP16/  UKN1  MTP  EF1β  **TIP41**  ACT11  PEPKR1  ACT2/7  UBQ10  HDC  TUA5  UKN2  TUB4  CYP | EF1β  MTP  **TIP41**  ACT11  UKN1  SKIP16  UBQ10  TUA5  ACT2/7  HDC  PEPKR1  UKN2  TUB4  CYP |  | SKIP16  UKN1  UKN2 | 4 |
| Linum  usitatissimum | ACT  CYC  EF1α  EF2  ETIF1  ETIF3E  ETIF3H  ETIF4F  ETIF5A  **GAPDH**  UBI  UBI2  TUA | EF1α/ETIF5A  UBI  GAPDH  ETIF1  UBI2  ETIF4F  ETIF3E  CYC  ACT  ETIF3H  EF2  TUA | GAPDH  UBI2  UBI  ETIF3E  EF1α  ETIF5A  ETIF4F  CYC  EF2  ETIF1  ACT  ETIF3H  TUA |  |  | 5 |
| Coffea arabica | POLY  ADH  ACT  GAPDH  RPL7  CSS  CYS  14-3-3 | Data not shown | Data not shown |  | GAPDH  14-3-3  RPL7 | 6 |
| Tomato | **GAPDH**  EFα1  TBP  RPL8  APT  DNAj  TUA  **TIP41**  **SAND**  **CAC**  **Expressed(AT4G33380)** | CAC/TIP4  TBP  SAND  Expressed  DNAj  APT  RPL8  GAPDH  EFα1  TUA | TIP4  CAC  Expressed  TBP  SAND  RPL8  APT  GAPDH/ DNAj  EFα1  TUA |  | CAC  TIP41  Expressed | 7 |
| Peach | 18SrRNA  ACT  CYP2  TEF2  **GAPDH**  PLA2  RP 11  RPL13  TUA  TUB  UBQ10 | TEF2/UBQ10  TUB  GAPDH  ACT  RP11  TUA  18SrRNA  PLA2  CYP2 | TEF2  RP11  TUB  UBQ10  CYP2  TUA  GAPDH  ACT  PLA2  18SrRNA | TEF2  UBQ10  RP 11  TUA  CYP2  GAPDH  TUB  ACT  PLA2  18SrRNA | TEF2  UBQ10  RP 11 | 8 |
| Soybean 2 | ACT11  CYP2  UBC2  EF1β  ACT2/7  G6PD  UBQ10  TUB  TUA  EF1α | ACT2/7/  TUA  EF1α  UBC2  EF1β  TUB  CYP2  ACT11  G6PD  UBC10 |  |  | EF1β  CYP2 | 9 |
| Poplar | ACT11  ACT2  CYP  TUA  TUB  UBQ  UBQ-L  ELF4B-L  EF1β  18SrRNA | - | - | - | UBQ  TUA | 10 |
| Brachopodium  distachyon | ACT7  EF1α  **GAPDH**  RCA  SamDC  TUA6  UBC18  Ubi4  Ubi10 | SamDC/ UBC18  GAPDH  Ubi4  RCA  ACT7  Ubi10  EF1α  TUA6 | Ubi4  SamDC  UBC18  GAPDH  ACT7  RCA  Ubi10  EF1α  TUA6 |  | UBC18 | 11 |
| Rice | ACT11  UBC  eEF-1α  **GAPDH**  β-TUB  eEF-4α  UBQ10  UBQ5  18SrRNA  25SrRNA | UBQ5/  eEF-1α  eEF-4α  25SrRNA  UBC  GAPDH  ACT2  UBQ10  18SrRNA  β-TUB |  |  | UBQ5  EF1α | 12 |
| Cichorium  intybus | ACT  TUB  NADHD  EF  H3  rRNA  **GAPDH** | ACT/ EF  rRNA  H3  TUB  NADHD  GAPDH | ACT  EF  rRNA  H3  TUB  NADHD  GAPDH | rRNA  ACT  EF  TUB  GAPDH  H3  NADHD | ACT  EF | 13 |
| Lolium perenne | eEF1α (h)  eEF1α (s)  TBP-1  eEF4α  YT521-B  eEF1α (m)  E2 | eEF1α (s)/ eEF1α (m)  TBP-1  E2  YT521-B  eEF4α  eEF1α (h) | eEF1α (s)  YT521-B  eEF1α (m)  eEF4α  TBP-1  E2  eEF1α (h) |  | EF1α (s)  YT521-B | 14 |
| Brachiaria brizantha | EF1α  EF4α  **GAPDH**  GDP  SUCOA  TUB  UBCE1, UBCE2  UBIBRA | UBCE1/  UBCE2  EF1  GAPDH  EF1α  SUCOA  UBIBRA  GDP |  |  | UBCE  EF1α | 15 |
| Triticum aestivum | *Ta54963*  *Ta54171*  Ta50503  *Ta2776*  *Ta54733*  *Ta54948*  *Ta30797*  *Ta35284*  *Ta54447*  *Ta54448*  Ta 22845  Ta659  **Ta30768**  *Ta53919*  Ta35497  Ta53937  *Ta53891*  *Ta45379*  *Ta1698*  Ta27771  Ta53964  *Ta54227*  Ta54280  *Ta54512*  *Ta4045*  *Ta53889*  *Ta53967*  Ta44405  Ta54238  Ta38797  *Ta55512*  *Ta2291* | Ta35284/  Ta22845  Ta54227  Ta2291  Ta53889  Ta4045  Ta2776  Ta53937  Ta54963  Ta53919  Ta54448  Ta54171  Ta54733  Ta54837  Ta54280  Ta54238  Ta53964  Ta50503  Ta55512  Ta25534  Ta54512  Ta1698  Ta30797  Ta54948  Ta30768  Ta54447  Ta35497  Ta27771  Ta54825  Ta53891  Ta44405  Ta38797 | Ta54227/  Ta2291  Ta2776  Ta35284  Ta53919  Ta53967  Ta54171  Ta54963  Ta54733  Ta4045  Ta54448  Ta22845  Ta53937  Ta53889  Ta54238  Ta54280  Ta50503  Ta53964  Ta54512  Ta55512  Ta25534  Ta1698  Ta30797  Ta54948  Ta30768  Ta54447  Ta35497  Ta27771  Ta54825  Ta53891  Ta44405  Ta38797 |  | Ta54227 (Cell div. control prot.)  Ta2291 (ADP-ribosylation factor)  Ta2776 (Similar to RNAse L inhibitor-like protein) | 16 |
| Petunia hybrida “Mitchell” | ACT  CYP  EF1α  **GAPDH**  RPS13  RAN1  **SAND**  TUB  UBQ | RAN1  SAND  UBQ  EF1α  RPS13  CYP  TUB  ACT  GAPDH | EF1α  CYP  RPS13  UBQ  ACT  SAND  TUB  GAPDH  RAN1 | CYP  EF1α  RPS13  ACT  UBQ  TUB  SAND  RAN1  GAPDH | EF1α  SAND | 17 |
| Petunia hybrida “V30” | ACT  CYP  EF1α  **GAPDH**  RPS13  RAN1  **SAND**  TUB  UBQ | RPS13  UBQ  RAN1  CYP  ACT  TUB  EF1α  SAND  GAPDH | UBQ  RAN1  ACT  GAPDH  RPS13  SAND  EF1α  TUB  CYP | CYP  EF1α  ACT  SAND  UBQ  GAPDH  RPS13  RAN1  TUB | CYP  RAN1 | 17 |
| Brassica napus | Act7  **SAND**  **UP1 (AT4G33380)**  **UP2 (AT4G26410)**  PP2A  **TIP41**  UBC9  UBC21 | UP1/UBC9  UBC21  TIP41  PP2A  SAND  UP2  Act7 |  |  | Vegetative tissues: UP1  UBC9  UBC21  TIP41  Maturing embryos:  ACT  UBC21  TIP41  PP2A | 18 |
| Eucalyptus globulus | 18S rRNA  ACT2  EF2  H2B  IDH  UBI  **SAND**  **TIP41**  *α-*Tubulin  **Ortholog AT4G33380**  EUC12 | IDH/SAND  EUC12  ACT2  *α-*Tubulin  H2B  EF2  TIP41  33380  UBI  18S rRNA | H2B  *α-*Tubulin  IDH  EUC12  TIP41  SAND  33380  EF2  ACT  UBI  18S rRNA |  | H2B  *α-*Tubulin | 19 |
| Pisum sativum | *GH720838*  *GH720843*  H3  PP2A  helicase  **GAPDH**  *α-*Tubulin  *β-*Tubulin  ACT  EF1α  18S rRNA | *PP2A/ β-*Tubulin  *GH720838*  ACT  H3  helicase  EF1α  *α-*Tubulin  *GH720843*  GAPDH  18S rRNA |  |  | Abiotic stress: PP2A/H3/b-tubulin/*GH720838*  Biotic stress: *GH720838*/b-tubulin/PP2A  Roots: *GH720838*/b-tubulin/PP2A  Leaves: EF-1a/b-tubulin (H3)  Roots and leaves non-stressed: *GH720838*/b-tubulin (H3) | 20 |
| Water lily | ACT11  **GAPDH**  EF1α  UBC16  RPS1  IF1  UBQ11  AP47 (CACS?) | AP47/ACT11  UBQ11  EF1α  UBC16  GAPDH  RPS1  IF1 | AP47  EF1α  ACT11  GAPDH  UBC16  UBQ11  RPS1  IF1 |  | AP47 (CACS)  ACT11 | 21 |
| Salvia miltiorrhiza | 18S rRNA  EF1α  EF1β  ACT  UBQ  *α-*Tubulin  *β-*Tubulin | ACT  UBQ  *α-*Tubulin  18S rRNA  EF1α |  |  | ACT  UBQ | 22 |

**References:**

1. Garg R, Sahoo A, Tyagi AK, Jain M (2010) Validation of internal control genes for quantitative gene expression studies in chickpea (Cicer arietinum L.). Biochem Biophys Res Commun 396(2):283-8.
2. Schmidt GW, Delaney SK (2010) Stable internal reference genes for normalization of real-time RT-PCR in tobacco (Nicotiana tabacum) during development and abiotic stress. Mol Genet Genomics 283(3):233-41.
3. Artico S, Nardeli SM, Brilhante O, Grossi-de-Sa MF, Alves-Ferreira M (2010) Identification and evaluation of new reference genes in Gossypium hirsutum for accurate normalization of real-time quantitative RT-PCR data. BMC Plant Biology 10:49.
4. Hu R, Fan C, Li H, Zhang Q, Fu YF (2009) Evaluation of putative reference genes for gene expression normalization in soybean by quantitative real-time RT-PCR. BMC Mol Biol 10:93.
5. Huis R, Hawkins S, Neutelings G (2010) Selection of reference genes for quantitative gene expression normalization in flax (Linum usitatissimum L.). BMC Plant Biology 10:71.
6. Cavallari CFBSF, Maluf MP, Maia IG (2009) Identification of suitable control genes for expression studies in Coffea Arabica under different experimental conditions. BMC Plant Biology, 10:1.
7. Exposito-Rodriguez M, Borges A, Borges-Perez A, Perez J (2008) Selection of internal control genes for quantitative real-time RT-PCR studies during tomato development process. BMC Plant Biology 8(1):131.
8. Tong Z, Gao Z, Wang F, Zhou J, Zhang Z (2009) Selection of reliable reference genes for gene expression studies in peach using real-time PCR. BMC Mol Biol 10:71.
9. Jian B, Liu B, Bi Y, Hou W, Wu C et al. (2009) Validation of internal control for gene expression study in soybean by quantitative real-time PCR. BMC Mol Biol 9(1):59.
10. Brunner AM, Yakovlev IA, Strauss SH (2004) Validating internal controls for quantitative plant gene expression studies. BMC Plant Biol 4:14.
11. Hong S, Yang MS, Xiang F, Park CM (2008) Exploring valid reference genes for gene expression studies in Brachypodium distachyon by real-time PCR. BMC Plant Biology 8:112.
12. Jain MNA, Tyagi AK, Khurana JP (2006) Validation of housekeeping genes as internal control for studying gene expression in rice by quantitative real-time PCR. Biochem Biophys Res Commun 345:646-651.
13. Maroufi A, Van Bockstaele E, De Loose M (2010) Validation of reference genes for gene expression analysis in chicory (Cichorium intybus) using quantitative real-time PCR. BMC Mol Biol 11:15.
14. Lee JM, Roche JR, Donaghy DJ, Thrush A, Sathish P (2010) Validation of reference genes for quantitative RT-PCR studies of gene expression in perennial ryegrass (Lolium perenne L.). BMC Mol Biol 11:8.
15. Silveira ED, Alves-Ferreira M, Guimarães LA, da Silva FR, Carneiro VT (2009) Selection of reference genes for quantitative real-time PCR expression studies in the apomictic and sexual grass Brachiaria brizantha. BMC Plant Biol 9:84.
16. Paolacci AR, Tanzarella OA, Porceddu E, Ciaffi M (2009) Identification and validation of reference genes for quantitative RT-PCR normalization in wheat. BMC Mol Biol 10:11.
17. Mallona I, Lischewski S, Weiss J, Hause B, Egea-Cortines M (2010) Validation of reference genes for quantitative real-time PCR during leaf and flower development in Petunia hybrida. BMC Plant Biol 10:4.
18. Chen X, Truksa M, Shah S, Weselake RJ (2010) A survey of quantitative real-time polymerase chain reaction internal reference genes for expression studies in Brassica napus. Anal Biochem 405(1):138-40.
19. de Almeida MR, Ruedell CM, Ricachenevsky FK, Sperotto RA, Pasquali G et al. (2010) Reference gene selection for quantitative reverse transcription-polymerase chain reaction normalization during in vitro adventitious rooting in Eucalyptus globulus Labill. [BMC Mol Biol](javascript:AL_get(this, 'jour', 'BMC Mol Biol.');) 11:73.
20. Die JV, Román B, Nadal S, González-Verdejo CI (2010) Evaluation of candidate reference genes for expression studies in Pisum sativum under different experimental conditions. Planta 232(1):145-53.
21. Luo H, Chen S, Wan H, Chen F, Gu C, Liu Z (2010) Candidate reference genes for gene expression studies in water lily. Anal Biochem 404(1):100-2.
22. Yang Y, Hou S, Cui G, Chen S, Wei J, Huang L (2010) Characterization of reference genes for quantitative real-time PCR analysis in various tissues of Salvia miltiorrhiza. Mol Biol Rep 37(1):507-13.
